# Thailand’s universal coverage scheme and its impact on health-seeking behavior

**DOI:** 10.1186/s40064-016-3665-4

**Published:** 2016-11-10

**Authors:** Seung Chun Paek, Natthani Meemon, Thomas T. H. Wan

**Affiliations:** 1 Department of Society and Health, Faculty of Social Sciences and Humanities, Mahidol University, Salaya, Phutthamonthon, Nakhon Pathom, 73170 Thailand; 2 Doctoral Program in Public Affairs, College of Health and Public Affairs, University of Central Florida, Orlando, FL 32816 USA

**Keywords:** Thailand, Universal Coverage Scheme, 30-Baht Scheme, Health-seeking behavior, Out-of-pocket payment

## Abstract

**Background:**

Thailand’s Universal Coverage Scheme (UCS) has improved healthcare access and utilization since its initial introduction in 2002. However, a substantial proportion of beneficiaries has utilized care outside the UCS boundaries. Because low utilization may be an indication of a policy gap between people’s health needs and the services available to them, we investigated the patterns of health-seeking behavior and their social/contextual determinants among UCS beneficiaries in the year 2013.

**Results:**

The study findings from the outpatient analysis showed that the use of designated facilities for care was significantly higher in low-income, unemployed, and chronic status groups. The findings from the inpatient analysis showed that the use of designated facilities for care was significantly higher in the low-income, older, and female groups. Particularly, for the low-income group, we found that they (1) had greater health care needs, (2) received a larger number of services from designated facilities, and (3) paid the least for both inpatient and outpatient services.

**Conclusions:**

This pro-poor impact indicated that the UCS could adequately respond to beneficiaries’ needs in terms of vertical equity. However, we also found that a considerable proportion of beneficiaries utilized out-of-network services, which implied a lack of universal access to policy services from a horizontal equity point of view. Thus, the policy should continue expanding and diversifying its service benefits to strengthen horizontal equity. Particularly, private sector involvement for those who are employed as well as the increased unmet health needs of those in rural areas may be important policy priorities for that. Lastly, methodological issues such as severity adjustment and a detailed categorization of health-seeking behaviors need to be further considered for a better understanding of the policy impact.

## Introduction

### Background

Thailand achieved universal health coverage in 2002 through the implementation of the Universal Coverage Scheme (UCS, also known as the 30-Baht Scheme). The UCS, as the main social health insurance program in the country, currently covers approximately 75% (approximately 47 million people) of the entire population, and it accounts for approximately 17% of the country’s total health expenditure^1^ (Antos 2007; Health Insurance System Research Office [HISRO] 2012).

Before the UCS in 2002, four health insurance programs were implemented, which were (1) the Civil Servant Medical Benefit Scheme (CSMBS), (2) the Social Security Scheme (SSS), (3) the Medical Welfare Scheme (MWS, also known as the Low-Income Card Scheme), and (4) the Voluntary Health Card Scheme (VHCS) (Towse et al. 2004). The CSMBS and SSS were health insurance programs for people in the formal employment sector, while the MWS and VHCS were for those in the informal employment sector.

Specifically, the CSMBS, as a fringe benefit, provided health insurance to government sector employees, their dependents (e.g., parents, spouses, and children), and retirees. The SSS was a compulsory health insurance program for private sector employees, and dependents and retirees were not covered by the scheme. The MWS, as a government subsidy program, was initially introduced for the poor in 1975. Later, its target population was expanded to include the elderly in 1992 and other vulnerable groups (e.g., children under the age of 12) in 1994. Lastly, the VHCS was a voluntary health insurance program for people who were not eligible for the other three programs. Each household could purchase one-year VHCS insurance coverage for 500 Baht (approximately US$15) (Damrongplasit and Melnick 2009).

The four insurance programs were intended to cover the entire population. However, both the MWS and VHCS programs faced operational issues, which caused approximately 30% (approximately 18 million people) to be uninsured by 2001. The MWS suffered from issues of mis-targeting due to difficulties with assessing the incomes of those working in the informal employment sector. A household survey in 2000 indicated that only 35% of all MWS beneficiaries met the MWS eligibility standards (HISRO 2012; NaRanong and NaRanong 2006; Suraratdecha et al. 2005; Tangcharoensathien et al. 2007). The VHCS suffered from issues of adverse selection. Possibly because of its voluntary nature, a study found that the presence of illness was positively associated with the purchase and utilization of the VHCS (Sakunphanit 2006; Supakankunti 2001).

Subsequently, the Thai government established the UCS program in 2002 by integrating the MWS and VHCS programs. Currently, three health insurance programs are implemented for the entire population, which are the CSMBS (9%), the SSS (16%), and the UCS (75%) (HISRO 2012). Table 1 provides the characteristics of the three health insurance programs in brief.

**Table 1:** Brief characteristics of three health insurance programs in Thailand *Source*: Thailand’s Universal Coverage Scheme: achievements and challenges: an independent assessment of the first 10 years, pp. 49, by Health Insurance System Research Office (HISRO) (2012), Nonthaburi, Thailand

*Civil Servant Medical Benefit Scheme (CSMBS, 9%): Per capita expenditure in 2010: US$ 367*	
Target population	Government employees, dependents including parents, spouse and up to two children (age < 20)
Financing source	General tax, noncontributory scheme
Payment method	Fee for service for outpatient services and conventional DRG for inpatient services
Health delivery	Free choice of public providers, no registration required
Benefits package	Slightly higher than SSS and UCS
*Social Security Scheme (SSS, 16%): Per capita expenditure in 2010: US$ 71*	
Target population	Private sector employees, excluding dependents
Financing source	Payroll tax financed, tri-partite contribution 1.5% of salary, equally by employer, employee and government
Payment method	Inclusive capitation for outpatient and inpatient services
Health delivery	Registered public and private competing contractors
Benefits package	Comprehensive: outpatient, inpatient, accident and emergency, high-cost care, with very minimal exclusion list; excludes prevention and health promotion
*Universal Coverage Scheme (UCS, 75%): Per capita expenditure in 2010: US$ 79*	
Target population	The rest of population not covered by SSS and CSMBS
Financing source	General tax
Payment method	Capitation for outpatient services and global budget plus DRG for inpatient services
Health delivery	Registered contractor provider, notably within the district health system
Benefits package	Similar to SSS, including prevention and health promotion for the whole population

The UCS provides a comprehensive benefits package for curative as well as rehabilitation services. Additionally, annual health check-ups and health promotion/disease prevention services are included in the package (Sakunphanit 2006). For health care delivery, the beneficiaries are required to receive health services from a designated facility, a primary care facility, as the gatekeeper to secondary and tertiary care. When the beneficiaries bypass their designated facilities, they are required to pay 100% out-of-pocket (OOP) costs (Limwattananon et al. 2007).

Capitation is the main provider payment method for outpatient services, while a global budget with diagnosis-related groups (DRGs) is the main payment method for inpatient services. The UCS is implemented solely through general tax revenue without contributions from beneficiaries (Antos 2007; HISRO 2012). When the policy was initially implemented in 2002, it required a 30-Baht copayment (approximately US$.90) for both outpatient and inpatient services except among vulnerable groups (i.e., the previous MWS beneficiaries included the poor, the elderly, and children under the age of 12).

Later, in 2006, the 30-Baht copayment requirement was abolished, and most services were free of charge until 2012 (Sakunphanit 2006). In 2012, the requirement was again reinstated with many exemption conditions. To explore the degree of the exemptions, we conducted a preliminary analysis and found that among the beneficiaries who used the UCS services, more than 80% received free services in 2013. Thus, the effects of the reinstated requirement did not appear to be significant.

### Significance of the study problem

Previous studies have investigated the impact of the UCS on health care utilization. Those studies consistently found that after the UCS implementation, the overall utilization of designated facilities increased significantly. First, using a trend analysis of health care utilization before and after the UCS, some studies found that the number of outpatient visits and/or inpatient admissions in designated facilities significantly increased after the UCS. Additionally, the increase was significantly higher in low-income groups (HISOR 2012; International Health Policy Program [IHPP] 2007a; Limwattananon et al. 2011; Tangcharoensathien et al. 2007, 2013).

Second, other studies have examined the changes in health-seeking behavior before and after the UCS. For outpatient care, the UCS increased outpatient service utilization in designated facilities, and simultaneously, it decreased informal care utilization such as over-the-counter (OTC) drugs and traditional medicine/healers^2^ (Limwattananon et al. 2013). For inpatient care, the UCS increased inpatient service utilization in designated facilities overall, and it shifted the utilization from non-designated facilities to designated facilities (Gruber et al. 2014). Additionally, these patterns occurred significantly more in low-income and older groups.

Furthermore, likely because the UCS increased the utilization of designated facilities that required no or minimal OOP payments and decreased the utilization of out-of-network services that required 100% OOP payments, several studies found that overall OOP health expenditures decreased significantly after the UCS, and this decrease also had an impact on the decreased incidence rate of household catastrophic health expenditures (HISRO 2012; IHPP 2007a; Limwattananon et al. 2007; Somkotra and Lagrada 2009).

Despite the positive effects, other studies have cited the insufficiency of the policy’s financing as a potential factor threatening service quality and access. An inadequate infrastructure as well as the brain drain resulting from skilled health workers moving from public to private sectors and rural to urban areas have been documented as issues on the supply side of the public sector (Antos 2007; IHPP 2007b; Sakunphanit 2006; Sakunphanit and Suwanrada 2011). Accordingly, those issues have caused service quality problems of the UCS such as long wait times and limited service access (Kirdruang 2011; Suwannarach et al. 2010).

For such quality problems, the Thai government has tried to encourage private sector involvement and expand benefits coverage. For benefits coverage expansion, the UCS has gradually expanded its benefits coverage from costly services/drugs to traditional Thai services/medicines^3^ (NHSO 2014; Rousseau 2014).

However, private sector involvement has been quite low. In 2007, the total numbers of public clinics (or health centers) and hospitals were 9758 and 1020 (156,494 beds), while the total numbers of private clinics and hospitals were 16,800^4^ and 318 (30,564 beds), respectively. Almost all public facilities joined the UCS program, while only 212 private facilities (152 clinics and 60 hospitals) joined the UCS program. Additionally, compared to 2013, the private sector involvement has not noticeably increased. In 2013, a total of 229 private facilities (191 clinics and 38 hospitals) joined the UCS program, and the facilities covered only approximately 5.7% of all UCS beneficiaries (HISRO 2012; National Health Security Office [NHSO] 2014; Sakunphanit and Suwanrada 2011).

Likely for this reason, a substantial proportion of UCS beneficiaries utilizes care outside their designated facilities. A study found that among all UCS beneficiaries, only 40% used the UCS services when they needed care, while the other 60% used services not covered by the UCS, which required 100% OOP payments (Kirdruang 2011). Some people might use informal care (e.g., buying OTC drugs or traditional medicine) due to accessibility problems of the UCS services (e.g., long wait times or transportation). Other people might use non-designated facilities due to acceptability problems of the UCS services (e.g., low quality or dissatisfaction with needed services).

On one hand, low utilization of the UCS services could be considered an appropriate use of health care if we assume that out-of-network services (either informal care or non-designated facilities) are relatively affordable and more accessible. On the other hand, low utilization may also mean that beneficiaries do not want to but are required to use out-of-network services due to individual issues (e.g., time constraints) and system issues (e.g., unavailability of needed services). In that sense, low utilization may be an indication of the policy gap between beneficiaries’ health needs and the services available to them. As such, understanding the differential utilization of health services (i.e., health-seeking behavior) and how the behavior is associated with social/contextual factors would be meaningful for current policy evaluations as well as future policy improvement.

In addition, the UCS is operated solely through general tax revenues without insurance contributions. Since all beneficiaries pay taxes, which are partially used for the UCS, its low utilization may also imply an inefficiency of policy financing, making people pay again for health care. As the stated objective of the UCS is to “entitle all Thai citizens to quality health care according to their needs regardless of their socioeconomic status” (HISRO 2012), it is important to understand where and why such policy gaps occur.

Therefore, the purpose of this study was to investigate health-seeking behaviors as well as their social/contextual determinants among UCS beneficiaries. Additionally, the study attempted to answer basic but fundamental questions—(1) who needs health care, (2) what types of health services are utilized, and (3) how much is the average OOP payment according to the type of service.

## Methods

### Data source and study sample

The study used Health and Welfare Survey (HWS) data from the year 2013. The HWS is a nationwide survey that annually collects data and is administered by the National Statistics Office (NSO) of Thailand. Using multi-stage random sampling, it represents a national cross section of all 76 provinces of Thailand, with approximately equal-sized samples from each province. The data includes comprehensive demographic and socioeconomic information at individual and/or household levels. Additionally, health utilization-related information (e.g., health insurance status and types of health services used) is available in the data.

The purpose of this study was to investigate factors associated with health-seeking behaviors among UCS beneficiaries. Thus, the study first selected the UCS beneficiaries. Then, among the beneficiaries, those who reported experiencing perceived illness during the survey period were selected again, and their health-seeking behaviors were analyzed.

Specifically, the HWS provides a set of questions regarding health-seeking behavior. For outpatient care, two questions were asked of respondents: (1) Have you felt sick during the 1 month prior to the survey date? and (2) If yes, what type of health services did you use? Likewise, for inpatient care, two similar questions were asked of respondents: (1) Have you been hospitalized during the 1 year prior to the survey date? and (2) If yes, what type of hospital did you stay in?

In the study, the beneficiaries who answered “yes” to question (2) were labeled as the “sick group,” while those who answered “no” to question (2) were labeled as the “non-sick group.” Additionally, the combined group of the sick and non-sick groups was labeled as the “overall group.” The sick group was used for the study’s main analysis (analysis of health-seeking behavior), while all three groups were used for the study’s descriptive analysis. In addition, the study sample was limited to adults over the age of 18 in order to reduce the impact of a potential bias due to juvenile health-seeking behavior, which is influenced by parents (Case and Paxson 2002; Isong et al. 2010). For the juvenile population (UCS beneficiaries under the age of 18), we also conducted the same analyses in the Appendix section.

The 2013 HWS originally included a total of 71,533 individuals and 23,697 households. Among the 71,533 individuals, 56,130 individuals (78.47%) were UCS beneficiaries, of which 40,614 individuals (72.36%) and 15,516 individuals (27.64%) were adults and juveniles, respectively. After data cleaning to eliminate missing and erroneous values, a total of 56,011 individuals (40,521 adults and 15,490 juveniles) was ultimately used as the overall group. In the overall group, the sick group for the outpatient analysis included 13,004 individuals (10,363 adults and 2641 juveniles), while the sick group for the inpatient analysis included 2859 individuals (2315 adults and 544 juveniles).

### Variable selection and measurement

The study used Andersen’s Healthcare Utilization Model to develop an analytical framework and select the study variables. Andersen’s Model classifies health utilization into three different factors, which are predisposing, enabling, and need-for-care factors (Aday and Andersen 1974; Andersen and Davidson 2001; Bradley et al. 2002; Lo and Fulda 2008).

This study included three demographic variables (age, gender, and marital status) as predisposing factors. For enabling factors, four socioeconomic variables (income, employment status, education, and dual coverage) were used as individual-level resources, while the variable region (urban and rural areas) was used as a community-level resource. Lastly, chronic status was used as the need-for-care factor.

For the dependent variable, the study defined health-seeking behavior as an action taken by individuals for the purpose of finding appropriate health care during their experience of perceived illness. Thus, the study used the sick group and classified health-seeking behaviors into four different types, which were (1) no care, (2) informal care, (3) designated facility care, and (4) non-designated facility care. “No care” indicated that the beneficiaries reported experiencing perceived illness but did not use any health services. “Informal care” was defined as the use of any health services not in designated or non-designated facilities. Specifically, the use of OTC drugs or traditional medicines/healers were included in this category. Lastly, “designated facility care” indicated that beneficiaries used health services in facilities designated by the UCS insurance, while “non-designated facility care” indicated that beneficiaries used health services in facilities not designated by the UCS insurance.

This study conducted outpatient and inpatient analyses separately. For the outpatient analysis, the dependent variable included four categories (no care, informal care, designated facility care, and non-designated facility care), while for the inpatient analysis, the dependent variable included two categories (designated facility care and non-designated facility care).

For income, the monthly household income was divided by the square root of the number of household members to obtain a standardized income per single-person household (Foster 2009). Since the income variable was skewed to the right, a log transformation was ultimately used. Age, specifically each individual’s age, was treated as a continuous variable, while gender was treated as a binary variable (male and female).

Marital status was measured as a categorical variable with three levels (single, married, and divorced/widowed/separated). Employment status was treated as a binary variable (yes and no). Education was measured as an ordinal variable with three levels (1 = primary school or below, 2 = middle or high school, and 3 = college or above). The study treated dual coverage as a binary variable (yes and no). If beneficiaries had dual insurance (e.g., UCS and private insurance), they were categorized in the yes group.

This study measured region as a binary variable (urban and rural). We defined municipalities as urban areas and non-municipalities as rural areas, according to the definition by the Thai NSO (NSO 2004). Lastly, chronic status was treated as a binary variable (yes and no). The Health and Welfare Survey defines 32 diseases^5^ as chronic/congenital diseases. If beneficiaries had any of the defined diseases, they were categorized in the yes group. If not, they were categorized in the no group.

### Statistical analysis

This study used binary logistic regression (BLR) and multinomial logistic regression (MLR) analyses. Since the dependent variable (health-seeking behavior) was a categorical variable, both BLR and MLR analyses were performed by setting designated facility care as a reference category. We used odds ratios and their statistical significance to assess the relationship between the dependent variable and the selected variables. If an odds ratio was greater than 1, it indicated a positive relationship. If an odds ratio was less than 1, it indicates a negative relationship (Hosmer and Lemeshow 2000). The statistical significance level was set at 0.05, and all of the analyses were performed using IBM SPSS Statistics Version 20.0 software.

## Results

### Descriptive statistics

Table 2 presents descriptive statistics from the outpatient analysis. For the overall group (n = 40,521), the average income and age were 9126.30 Baht and 47.44 years, respectively. In the group, 46.26% were male, and 53.74% were female. For educational level, 67.58% had completed primary school or below, 28.12% had completed middle or high school, and 4.30% had completed college or above. For marital status, 17.32, 66.94, and 15.74% were single, married, and divorced/widowed/separated, respectively. For employment status, 73.36% were employed, while 26.64% were not employed. For dual coverage, 4.58% had dual insurance, while 95.42% had only the UCS insurance. For chronic status, 24.67% had at least one of the defined chronic/congenital diseases. Lastly, for region, 51.47% lived in urban areas, while 48.53% lived in rural areas.

**Table 2:** Descriptive statistics for the study variables (outpatient analysis, age 18+)

Variables	Sick group (n = 10,363)		Non-sick group (n = 30,158)		Overall group (n = 40,521)	
	Mean or percent	Std. Dev	Mean or percent	Std. Dev	Mean or percent	Std. Dev
*Health*-*seeking behavior*						
No care	11.22%					
Informal care	18.89%					
Designated facility care	59.65%					
Non-designated facility care	10.23%					
*OOP payment*						
Informal care	81.32	149.84				
Designated facility care	55.44	448.40				
Non-designated facility care	777.40	1472.00				
Income	8244.68	13,678.83	9429.25	11,401.58	9126.30	12,036.01
Income (log transformed)	8.54	1.27	8.76	1.08	8.70	1.13
Age	56.44	16.09	44.34	15.88	47.44	16.79
*Gender*						
Male	39.26%		48.67%		46.26%	
Female	60.74%		51.33%		53.74%	
*Education*						
Primary school or below	81.84%		62.68%		67.58%	
Middle or high school	15.78%		32.36%		28.12%	
College or above	2.38%		4.96%		4.30%	
*Marital status*						
Single	10.20%		19.77%		17.32%	
Married	64.36%		67.83%		66.94%	
Divorced/widowed/separated	25.44%		12.40%		15.74%	
*Employment status*						
Yes	61.68%		77.38%		73.36%	
No	38.32%		22.62%		26.64%	
*Dual coverage*						
Yes	4.32%		4.67%		4.58%	
No	95.68%		95.33%		95.42%	
*Chronic status*						
Yes	65.49%		10.64%		24.67%	
No	34.51%		89.36%		75.33%	
*Region*						
Urban	47.86%		52.71%		51.47%	
Rural	52.14%		47.29%		48.53%	

Std. Dev, standard deviation; OOP Payment, out-of-pocket payment; Median (interquartile range Q1–Q3) of out-of-pocket payment for informal care = 50 (20–100); Median (interquartile range Q1–Q3) of out-of-pocket payment for designated facility care = 0 (0–0); Median (interquartile range Q1–Q3) of out-of-pocket payment for non-designated facility care = 320 (200–600); Median (interquartile range Q1–Q3) of income for sick group = 5813.78 (3265.99–9899.49)

In the overall group, approximately 25.57% reported experiencing perceived illness (sick group, n = 10,363). In the sick group, 59.65% received care from a designated facility. Additionally, 18.89 and 10.23% received informal care and care from a non-designated facility, respectively. Furthermore, 11.22% did not use any services even though they reported experiencing perceived illness. For OOP payment, beneficiaries who received care from a non-designated facility paid 777.40 Baht on average (median = 320 Baht). For informal care and designated facility care, beneficiaries paid on average 81.32 Baht (median = 50 Baht) and 55.44 Baht (median = 0 Baht), respectively.

Table 2 also presents additional descriptive statistics for the non-sick group for comparison purposes. We conducted both a *t* test and Chi square test (not presented in the table) to compare the groups. The results indicated that the sick group included a higher proportion of low-income, older, female, unemployed, and chronically ill individuals. For education, a higher proportion of people had a primary school level of education or below, and a lower proportion had a middle or high school level of education or college level or above in the sick group. For marital status, the sick group included a higher proportion of single and divorced/widowed/separated individuals and a lower proportion of married individuals. Lastly, for region, the sick group had more people living in rural areas.

Table 3 presents descriptive statistics from the inpatient analysis. In the overall group, approximately 5.71% reported experiencing perceived illness (sick group, n = 2315). In the sick group, 91.75% received care from a designated facility, while 8.25% received care from a non-designated facility. For OOP payment and length of stay, beneficiaries who received care from a designated facility paid 185.37 Baht (median = 0 Baht) per day and stayed for 6.27 days (median = 3 days) on average. Meanwhile, beneficiaries who received care from a non-designated facility paid 6614.80 Baht (median = 4285.71 Baht) per day and stayed for 4.41 days (median = 3 days) on average.

**Table 3:** Descriptive statistics for the study variables (inpatient analysis, age 18+)

Variables	Sick group (n = 2135)		Non-sick group (n = 38,206)		Overall group (n = 40,521)	
	Mean or percent	Std. Dev	Mean or percent	Std. Dev	Mean or percent	Std. Dev
*Health*-*seeking behavior*						
Designated facility care	91.75%					
Non-designated facility care	8.25%					
*OOP payment (per day)*						
Designated facility care	185.37	958.18				
Non-designated facility care	6614.80	11,797.00				
*Length of stay*						
Designated facility care	6.27	9.81				
Non-designated facility care	4.41	8.77				
Income	8421.74	20,101.62	9168.99	11,363.87	9126.30	12,036.01
Income (log transformed)	8.55	1.21	8.71	1.13	8.70	1.13
Age	51.39	18.99	47.20	16.61	47.44	16.79
*Gender*						
Male	38.57%		46.73%		46.26%	
Female	61.43%		53.27%		53.74%	
*Education*						
Primary school or below	72.10%		67.31%		67.58%	
Middle or high school	24.06%		28.36%		28.12%	
College or above	3.84%		4.33%		4.30%	
*Marital status*						
Single	8.90%		17.83%		17.32%	
Married	70.58%		66.72%		66.94%	
Divorced/widowed/separated	20.52%		15.45%		15.74%	
*Employment status*						
Yes	55.51%		74.45%		73.36%	
No	44.49%		25.55%		26.64%	
*Dual coverage*						
Yes	6.70%		4.45%		4.58%	
No	93.30%		95.55%		95.42%	
*Chronic status*						
Yes	50.93%		23.08%		24.67%	
No	49.07%		76.92%		75.33%	
*Region*						
Urban	50.15%		51.55%		51.47%	
Rural	49.85%		48.45%		48.53%	

Std. Dev, standard deviation; OOP Payment, out-of-pocket payment; Median (interquartile range Q1–Q3) of out-of-pocket payment for designated facility care = 0 (0–6); Median (interquartile range Q1–Q3) of out-of-pocket payment for non-designated facility care = 4285.71 (66.67–8333.17); Median (interquartile range Q1–Q3) of length of stay for designated facility care = 3 (2–6); Median (interquartile range Q1–Q3) of length of stay for non-designated facility care = 3 (2–4); Median (interquartile range Q1-Q3) of income for sick group = 5773.503 (3265.99–9814.96)

Table 3 also provides descriptive statistics for the non-sick group for comparison purposes. The results from both the *t* test and Chi square test indicated that the sick group included a higher proportion of low-income, older, female, unemployed, dual coverage, and chronically ill individuals. For education, the sick group included a higher proportion of people with a primary school level of education or below and a lower proportion of those with a middle or high school level of education. However, the proportion of individuals with a college level of education or above was not significantly different between the sick and non-sick groups. For marital status, the sick group included a lower proportion of single individuals and a higher proportion of married and divorced/widowed/separated individuals. Lastly, region was not significantly different between the sick and non-sick groups.

### Results of health-seeking behavior analysis

Table 4 presents the results from the MLR outpatient analysis. The results of the Pearson’s goodness-of-fit test for the MLR model did not present a lack of fit with a *p* value greater than 0.05. In the model for informal care (vs. designated facility care), a significant relationship was found in six variables (income, age, marital status, employment status, dual coverage, and chronic status). Income was positively related to informal care with an odds ratio equal to 1.195. It indicated that people with higher incomes tended to use informal care, while those with lower incomes tended to use designated facility care. Age was negatively associated with informal care. The odds ratio (0.995) meant that younger adults were more likely to use informal care than older adults.

**Table 4:** Results of MLR outpatient analysis (Sick group, age 18+) (n = 10,363)

Variables	No care		Informal care		Non-designated facility care	
	OR	95% CI	OR	95% CI	OR	95% CI
Income (log transformed)	0.985	(0.938, 1.033)	1.195	(1.130, 1.264)*	1.324	(1.226, 1.431)*
Age	0.995	(0.990, 1.001)	0.995	(0.990, 0.999)*	0.994	(0.989, 1.000)
Gender female (vs. Male)	0.921	(0.803, 1.056)	1.021	(0.907, 1.149)	1.209	(1.047, 1.396)*
Education						
College or above	1.000		1.000		1.000	
Primary school or below	0.738	(0.475, 1.146)	0.874	(0.600, 1.273)	0.521	(0.357, 0.758)*
Middle or high school	0.817	(0.520, 1.284)	0.945	(0.644, 1.387)	0.683	(0.465, 1.003)
Marital status						
Married	1.000		1.000		1.000	
Single	1.312	(1.048, 1.644)*	1.049	(0.858, 1.283)	0.985	(0.774, 1.254)
Divorced/widowed/separated	1.169	(0.983, 1.389)	1.238	(1.067, 1.436)*	0.991	(0.827, 1.187)
Employment status yes (vs. no)	1.134	(0.967, 1.329)	1.151	(1.004, 1.320)*	1.236	(1.052, 1.454)*
Dual coverage yes (vs. no)	1.798	(1.287, 2.512)*	1.626	(1.220, 2.167)*	3.793	(2.931, 4.908)*
Chronic status yes (vs. no)	0.200	(0.173, 0.230)*	0.114	(0.101, 0.129)*	0.425	(0.366, 0.495)*
Region rural (vs. urban)	1.513	(1.322, 1.731)*	0.994	(0.887, 1.114)	0.950	(0.829, 1.090)
*Pearson’s goodness*-*of*-*fit test*						
Chi square (*df*) = 30,567.910 (30,270)						
*p* value = 0.113						

Reference = designated facility care

*OR* odds ratio, *95% CI* 95% confidence interval

* *p* value < 0.05

For marital status, the category of divorced/widowed/separated was positively associated with informal care. It meant that divorced/widowed/separated individuals were more likely to use informal care than single individuals. For employment status, the employed group tended to use informal care, while the unemployed group tended to use designated facility care. For dual coverage, people with dual insurance coverage were more likely to use informal care than those with only UCS insurance. Lastly, chronic status was negatively related to informal care. Beneficiaries without any defined chronic/congenital diseases tended to use informal care, while those with a defined chronic/congenital disease tended to use designated facility care.

In the model for non-designated facility care (vs. designated facility care), a significant relationship was found in six variables (income, gender, educational level, employment status, dual coverage, and chronic status). For income, similar to the results from the informal care model above, beneficiaries with higher incomes were more likely to receive care from a non-designated facility than those with lower incomes. For gender, the odds ratio (1.203) indicated that females were more inclined to receive care from a non-designated facility than males.

For education, the use of a non-designated facility for care was significantly higher in individuals with a college level of education or above than individuals with a primary school level of education or below. However, a significant difference was not found between those with a college level of education or above and those with a middle or high school level of education. For employment status, employed individuals were more inclined to receive care from a non-designated facility than unemployed individuals. For dual coverage and chronic status, people with dual insurance coverage or without any chronic/congenital diseases tended to receive care from a non-designated facility.

Lastly, in the model for no care (vs. designated facility care), four variables (marital status, chronic status, dual coverage, and region) were statistically significant. For marital status, single status was positively related to receiving no care. This meant that single individuals were less likely to use health services from a designated facility than married individuals when experiencing perceived illness. For dual coverage and chronic status, people with dual insurance coverage or without any chronic/congenital diseases were less inclined to use services. For region, people in rural areas were less likely to use services from a designated facility than those in urban areas.

Table 5 presents the results of the BLR inpatient model. The Hosmer–Lemeshow goodness-of-fit test did not show a lack of fit for the model with a *p* value greater than 0.05. In the BLR model, six variables were statistically significant, which were income, age, gender, education, marital status, and dual coverage. For income and age, the higher income group and older group were more likely to receive care from a non-designated facility than the low-income group and younger group, compared to receiving care from a designated facility. For gender, females were more inclined to receive care from a non-designated facility than males.

**Table 5:** Results of BLR inpatient analysis (sick group, age 18+) (n = 2135)

Variables	Non-designated facility care	
	OR	95% CI
Income (log transformed)	1.980	(1.587, 2.472)*
Age	1.017	(1.003, 1.032)*
Gender female (vs. male)	1.576	(1.072, 2.316)*
Education		
College or above	1.000	
Primary school or below	0.240	(0.128, 0.450)*
Middle or high school	0.382	(0.208, 0.700)*
Marital status		
Married	1.000	
Single	2.125	(1.247, 3.623)*
Divorced/widowed/separated	0.756	(0.439, 1.302)
Employment status yes (vs. no)	1.076	(0.737, 1.572)
Dual coverage yes (vs. no)	10.981	(7.199, 16.749)*
Chronic status yes (vs. no)	0.667	(0.443, 1.006)
Region rural (vs. urban)	0.853	(0.600, 1.211)
*Hosmer*–*Lemeshow goodness*-*of*-*fit test*		
Chi square (*df*) = 8.948 (8)		
*p* value = 0.347		

Reference = designated facility care

*OR* odds ratio, *95% CI* 95% confidence interval

* *p* value < 0.05

For education, the higher education group was more likely to receive care from a non-designated facility. Specifically, people with a college level of education or above were more likely to receive care from a non-designated facility than those with a primary school level of education or below and middle or high school levels of education. For marital status, single individuals more often received care from a non-designated facility than the married individuals. Lastly, for dual coverage, the group with dual insurance coverage received significantly more care from a non-designated facility.

## Discussion

The study investigated the patterns of health-seeking behavior among UCS beneficiaries in the year 2013. By classifying health-seeking behavior into four categories for the outpatient analysis (no care, informal care, designated facility care, non-designated facility care) and two categories for the inpatient analysis (designated facility care and non-designated facility care), we examined how the behavior was influenced by selected social/contextual factors.

The study findings indicated that the UCS increased the use of designated facilities in vulnerable groups. Specifically, the outpatient analysis showed that receiving care from designated facilities was significantly higher in low-income, unemployed, and chronically ill groups. The inpatient analysis showed that receiving care from designated facilities was significantly higher in low-income, older, and female groups.

This pro-poor impact could be explained using the following equity perspective: vertical and horizontal equity. First, to some degree, the UCS could adequately meet the assumption that vertical equity in access to health services implies that people with greater health care needs must receive more health services than those with fewer needs.

Excessive OOP expenditures have been cited as one of the significant barriers to health access for people, particularly low-income and socially vulnerable groups. When a health system becomes more dependent on OOP expenditures, it can cause catastrophic health expenditures for these people. Accordingly, catastrophic expenditures can drive them back into poverty. Thus, vertical equity is one of the important considerations of health systems operations (OECD 2013; WHO 2013). Since the UCS significantly increased the use of designated facilities for care, which required no or less OOP payments for low-income groups who also had greater health care needs, the policy could have adequately responded to beneficiaries’ needs in terms of vertical equity, for which the elimination of financial barriers to access to health services was a major factor.

However, the findings showed that a considerable proportion of beneficiaries used out-of-network services (e.g., approximately 37% of the beneficiaries using outpatient services used either informal care or care from non-designated facilities). In fact, the use of out-of-network services could have been considered an appropriate use of health services if we assumed that people had different preferences and choices. However, the premise of horizontal equity is the universality of access to health services. That is, beneficiaries who have equal health care needs should receive equal treatment, regardless of their socioeconomic situations. This means that the UCS policy must enable all beneficiaries to access their health care benefits under the scheme when needed.

Thus, the evidence of high utilization of out-of-network services could imply a lack of universal access to services provided under the UCS policy. Accessibility issues (e.g., long wait times or transportation) and/or acceptability issues (e.g., low quality or dissatisfaction with needed services) might discourage some groups of beneficiaries (e.g., higher-income groups or employed groups) and prompt them to seek care outside the UCS boundaries. These might be some of the factors hampering universal access and thus horizontal equity.

The stated objective of the UCS is to “entitle all Thai citizens to quality health care according to their needs regardless of their socioeconomic status” (HISRO 2012), which implies that the UCS should not be a selective welfare policy that targets only vulnerable populations but a universal welfare policy that targets everyone. Additionally, considering that the UCS is a tax-based policy, improving its universality should be one of the critical aims for future policy improvement.

In that regard, we aim to discuss possible reasons and policy interventions related to the high utilization of out-of-network services (or low utilization of UCS services) from the perspective of OOP payments and affordability. The study’s findings showed that for outpatient care, people who used designated facilities paid 55.44 Baht on average. Those who used informal care and non-designated facilities paid 81.32 and 777.40 Baht on average, respectively. For inpatient care, people who used designated facilities paid 185.37 Baht (per day), while those who used non-designated facilities paid 6614.80 Baht (per day) on average.

For informal care versus designated facility care, the differences in average OOP payments did not seem to be significant. Compared to individual average income (approximately 8400 Baht), the average OOP payment for informal care was less than 1% of the average income, which seemed affordable.

However, between designated facility care versus non-designated facility care, the average OOP payments were significantly different. Specifically, the average OOP payment for outpatient services from non-designated facilities was approximately 10% of the average income, and the average OOP payment for inpatient services from non-designated facilities care was approximately 80% of the average income. Both amounts could hardly be considered affordable for most beneficiaries.

In that sense, private sector involvement would be highly prioritized to address the issue of low utilization. The findings from the outpatient analysis showed that employed individuals depended more on non-designated facilities for care. Since employed individuals may have had less access to designated facilities during the day in general, it was possible that they were compelled to use private facilities after their work hours. As mentioned previously, a total of 229 private facilities (191 clinics and 38 hospitals) joined the UCS program in 2013, which covered only approximately 5.7% of all UCS beneficiaries. Compared to the total number of private facilities (16,800 clinics and 318 hospitals in 2007), 229 private facilities was quite a small number. Along with the previous finding that more than 80% of health services were sought in private clinics (Kirdruang 2011), private sector involvement (particularly private clinic involvement) would be one of the important priorities to address the issue of low utilization.

In addition, affordable costs make the use of informal care more attractive and efficient for those who might have accessibility problems with designated facilities. There may be patients with minor symptoms who desire rapid treatment or medication, and longer wait times or transportation difficulties likely prompt them to opt for informal care. In that sense, private sector involvement as well as benefits coverage expansion to include frequently used medicines may be potential interventions for informal care users.

The higher cost of receiving care from non-designated facilities (particularly inpatient care) likely implies acceptability problems such as low quality or dissatisfaction with needed services at designated facilities. Recipients of care from non-designated facilities may be patients with severe conditions who accordingly need advanced quality services, but these acceptability problems may push them out to non-designated facilities. In that sense, the policy needs to continue improving service quality by expanding and diversifying its benefits according to social needs and consensus. Nevertheless, the actual reasons for these different health-seeking behaviors could be more diverse and significantly differ by social and contextual factors. Because different reasons require different policy interventions, the actual reasons need to be systematically investigated for future policy improvement.

The low utilization of UCS services was found to improve from 2007 to 2013. Specifically, the use of designated facility care increased from 42.78 to 57.54%, the use of informal care decreased from 28.53 to 19.12%, the use of non-designated facility care decreased from 23.35 to 12.90%, and no care increased from 5.34 to 10.44%^6^ (Kirdruang 2011). As mentioned previously, we did not notice a significant increase in private sector involvement from 2007 to 2013, thus, this improvement may be partially caused by the expanded benefits coverage of the UCS. Thus, future studies need to examine the impact of the benefits coverage expansion on the use of designated facilities for care. Additionally, other possible confounding factors (e.g., changes in beneficiaries’ perceptions of UCS service quality) should be further measured and investigated for a better understanding of this improvement.

In addition, we found that the proportion of people who did not use any health services despite reporting perceived illness increased almost twofold from 5.34% in 2007 to 10.44% in 2013. Furthermore, we noticed some changes in the factors associated with this unmet health need. Specifically, previous findings indicated that in 2007, people in rural areas had significantly higher utilization of designated facilities for care and lower unmet health needs. However, this study found that in 2013, the use of designated facilities for care was not significantly different between rural and urban areas. Additionally, people in rural areas had higher unmet needs (Kirdruang 2011; Limwattananon et al. 2013).

This non-significant relationship of designated facility care utilization between rural and urban areas may have been because either the utilization of UCS services in urban areas relatively increased or the utilization in rural areas relatively decreased. However, the significant increase in unmet needs in rural areas implied the possibility that the utilization of UCS services in rural areas could relatively decrease. Thus, such changes and related factors need to be further explored. This study used a simplistic categorization of region (i.e., urban and rural areas) and did not use a regional distribution of health resources (e.g., the number of health personnel and health facilities by each province or district). We expect that such information would be useful to understand the increased unmet need as well as the regional variation in health-seeking behavior, particularly for people living in remote areas.

Lastly, we would like to mention several limitations in the study. First, the simplistic categorization of health-seeking behavior (e.g., no care, informal care, designated facility care, and non-designated facility care) might not accurately capture the policy impact. Particularly, this study defined both OTC drugs and traditional medicines/healers as the category of informal care. However, certain types of traditional medicines are available in the formal care sector in both designated and non-designated facilities. For instance, some larger designated facilities have their own traditional medicine clinics/departments that operate as independent for-profit units requiring 100% OOP payments.

Additionally, some traditional medicines and doctors are available in licensed traditional medicine clinics, which could be considered non-designated facilities. The HWS data does not specify such information in detail, and therefore, survey respondents could only choose between either herbal/traditional medicine or designated/non-designated facilities. For such issues, the study followed a similar process to previous studies (Kirdruang 2011; Limwattananon et al. 2013).

We initially attempted to use separate categories for OTC drugs and traditional medicines/healers. However, a small sample size (i.e., only 156 beneficiaries used traditional medicines/healers among 13,004 beneficiaries who reported experiencing perceived illness) could not provide a stable estimation in the analysis. Thus, we ultimately combined OTC drugs and traditional medicines/healers and used the combination as the category of informal care in the analysis.

Second, health-seeking behavior varies according to the different severity level of the illness/sickness in general. However, this study was unable to adjust for severity in the analysis because the HWS data, as survey data, provided comprehensive socioeconomic information rather than clinical information. Thus, the policy impact found in this study might have been overestimated or underestimated. If HWS data could be merged with medically related data such as medical claims data or hospital administration data in future studies, it would offer more precise estimations of the policy impact.

Third, the study focused mainly on exploring patterns of health-seeking behavior rather than conducting a cost analysis. A cost-effectiveness or efficiency analysis would be important to evaluate the policy impact from an efficiency point of view. Additionally, health outcome evaluation research would provide additional meaningful insight on the policy impact.

Lastly, the HWS data represented a snapshot of individual health-seeking behavior over only a 1-month period for outpatient care and a 1-year period for inpatient care prior to the survey date, rather than a complete picture between surveys. Thus, a longitudinal analysis to explore changes in health-seeking behavior as well as their social/contextual determinants over time are encouraged to investigate the long-term effects of the policy.

## Conclusions

This study investigated patterns of health-seeking behavior and their social/contextual determinants among UCS beneficiaries. The study findings showed that the UCS was adequately responsive to the needs of beneficiaries from a vertical equity perspective. Particularly, for the low-income group, we found that they (1) had more health care needs, (2) received a larger number of services from designated facilities, and (3) paid the least for both inpatient and outpatient services. Nevertheless, the study also found that a substantial proportion of beneficiaries still utilized out-of-network services, which could imply a lack of universal access to policy services from a horizontal equity point of view. Thus, the policy should continue expanding and diversifying its service benefits to strengthen horizontal equity. Particularly, private sector involvement for those who are employed as well as the increased unmet health needs of those in rural areas may be important policy priorities for that. Lastly, methodological issues such as severity adjustment and a detailed categorization of health-seeking behaviors need to be further considered to better understand the policy impact.

## Appendix 1

See Table 6.

**Table 6:** Descriptive statistics for the study variables (outpatient analysis, age 18−)

Variables	Sick group (n = 2641)		Non-sick group (n = 12,849)		Overall group (n = 15,490)	
	Mean or percent	Std. Dev	Mean or percent	Std. Dev	Mean or percent	Std. Dev
*Health*-*seeking behavior*						
No care	7.35%					
Informal care	19.99%					
Designated facility care	49.26%					
Non-designated facility care	23.40%					
*OOP payment*						
Informal care	76.21	104.50				
Designated facility care	43.01	399.61				
Non-designated facility care	397.04	601.32				
Income	8792.35	10,932.72	8399.29	10,003.72	8466.31	10,168.82
Income (log transformed)	8.73	0.90	8.63	1.15	8.65	1.11
Age	6.98	4.94	9.43	4.98	9.01	5.06
*Gender*						
Male	50.74%		50.57%		50.60%	
Female	49.26%		49.43%		49.40%	
*Dual coverage*						
Yes	4.92%		4.08%		4.22%	
No	95.08%		95.92%		95.78%	
*Region*						
Urban	46.61%		51.82%		50.93%	
Rural	53.39%		48.18%		49.07%	

Std. Dev, standard deviation; OOP Payment, out-of-pocket payment; Median (interquartile range Q1–Q3) of out-of-pocket payment for informal care = 50 (20–100); Median (interquartile range Q1–Q3) of out-of-pocket payment for designated facility care = 0 (0–0); Median (interquartile range Q1–Q3) of out-of-pocket payment for non-designated facility care = 300 (190–450); Median (interquartile range Q1–Q3) of income for sick group = 6368.67 (3868.25–10,392.30)

## Appendix 2

See Table 7.

**Table 7:** Descriptive statistics for the study variables (inpatient analysis, age 18−)

Variables	Sick group (n = 544)		Non-sick group (n = 14,946)		Overall group (n = 15,490)	
	Mean or percent	Std. Dev	Mean or percent	Std. Dev	Mean or percent	Std. Dev
*Health*-*seeking behavior*						
Designated facility care	90.63%					
Non-designated facility care	9.38%					
*OOP payment (per day)*						
Designated facility care	153.85	628.75				
Non-designated facility care	1495.13	2383.00				
*Length of stay*						
Designated facility care	4.45	6.11				
Non-designated facility care	3.33	1.66				
Income	8144.05	8429.73	8478.04	10,226.59	8466.31	10,168.82
Income (log transformed)	8.61	1.11	8.65	1.11	8.65	1.11
Age	7.83	5.44	9.06	5.04	9.01	5.06
*Gender*						
Male	54.41%		50.46%		50.60%	
Female	45.59%		49.54%		49.40%	
*Dual coverage*						
Yes	11.40%		3.96%		4.22%	
No	88.60%		96.04%		95.78%	
*Region*						
Urban	44.85%		51.15%		50.93%	
Rural	55.15%		48.85%		49.07%	

Std. Dev, standard deviation; OOP Payment, out-of-pocket payment; Median (interquartile range Q1–Q3) of out-of-pocket payment for designated facility care = 0 (0–0); Median (interquartile range Q1–Q3) of out-of-pocket payment for non-designated facility care = 83.33 (0–2500); Median (interquartile range Q1–Q3) of length of stay for designated facility care = 3 (2–5); Median (interquartile range Q1–Q3) of length of stay for non-designated facility care = 3 (2–4); Median (interquartile range Q1–Q3) of income for sick group = 6000 (3535.53–9800.44)

## Appendix 3

See Table 8.

**Table 8:** Results of MLR outpatient analysis (sick group, age 18−) (n = 2641)

Variables	No care		Informal care		Non-designated facility care	
	OR	95% CI	OR	95% CI	OR	95% CI
Income (log transformed)	1.051	(0.886, 1.246)	1.337	(1.172, 1.526)*	1.795	(1.570, 2.051)*
Age	1.080	(1.047, 1.113)*	1.079	(1.057, 1.101)*	0.969	(0.949, 0.990)*
Gender female (vs. male)	0.877	(0.646, 1.189)	1.028	(0.836, 1.264)	1.153	(0.946, 1.405)
Dual coverage yes (vs. no)	1.276	(0.602, 2.705)	0.961	(0.559, 1.655)	2.174	(1.409, 3.356)*
Region rural (vs. urban)	1.194	(0.871, 1.636)	0.639	(0.518, 0.788)*	0.716	(0.586, 0.876)*
*Pearson goodness*-*of*-*fit test*						
Chi square (*df*) = 8389.083 (7281)						
*p* value ≤ 0.001						

Reference = designated facility care

*OR* odds ratio, *95% CI* 95% confidence interval

* *p* value < 0.05

## Appendix 4

See Table 9.

**Table 9:** Results of BLR inpatient analysis (sick group, age 18−) (n = 544)

Variables	Non-designated facility care	
	OR	95% CI
Income (log transformed)	2.499	(1.436, 4.346)*
Age	0.935	(0.867, 1.009)
Gender female (vs. male)	1.281	(0.594, 2.763)
Dual coverage yes (vs. no)	38.003	(16.953, 85.188)*
Region rural (vs. urban)	1.360	(0.612, 3.021)
*Hosmer*–*Lemeshow goodness*-*of*-*fit test*		
Chi square (*df*) = 5.230 (8)		
*p* value = 0.732		

Reference = designated facility care

*OR* odds ratio, *95% CI* 95% confidence interval

* *p* value < 0.05

## Footnotes

1 Thailand’s total health expenditure is composed of six different funding sources—(1) UCS (17%), (2) CSMBS (12%), (3) SSS (8%), (4) private insurance (9%), (5) OOP spending (27%), and (6) other public spending (27%). “Other public spending” which is for operating and staffing the system of public health facilities is financed by the Ministry of Public Health (72.5%), local governments (15%), and other ministries (12.5%) (Antos 2007).

2 Thailand has its own system of traditional medicine that covers medical practices (e.g., diagnosis and treatment), pharmacy practices (e.g., use of medicinal plants, animals or minerals), midwifery and traditional massage. In the past, traditional Thai medicine had been generally offered by local or non-institutional healers at their homes and religious institutions (Chokevivat and Chuthaputti 2005; Peltzer et al. 2016).

3 Over the past decades, the national health development plan (2007–2011) launched an agenda to integrate traditional Thai medicine into the public health care system. In 2008, traditional and alternative medicine accounted for 0.05% of the global UC budget (NHSO 2008). The budget per capita for the use of traditional Thai medicine has increased continuously. The Department of Thai Traditional and Alternative Medicine (2011) estimated that 10% of Thai people seeking care in public health facilities received traditional Thai medicine.

4 Most private clinics belong to medical doctors who work in public facilities, and the doctors operate their own clinics after office hours (Sakunphanit and Suwanrada 2011).

5 1. diabetes; 2. thyroid/goiter; 3. hypertension; 4. heart disease (cardiomegaly/cardiomyopathy/stenosis heart disease/valvular heart disease/coronary artery disease); 5. hyperlipidemias/hypercholesterolemia; 6. stroke; 7. cancer (lung/liver/colon cancer etc.); 8. alcoholism; 9. depression/other mental disorders; 10. dementia; 11. epilepsy/grand mal seizure; 12. cataracts/glaucoma; 13. hearing loss; 14. asthma/emphysema; 15. tuberculosis; 16. allergies; 17. cirrhosis; 18. renal failure/nephrosis; 19. benign prostatic hyperplasia/prostatitis; 20. gallstones; 21. urinary bladder stones (kidney/bladder); 22. gout/rheumatoid; 23. chronic knee pain/osteoarthritis; 24. chronic back pain/chronic neck pain; 25. AIDS; 26. poliomyelitis; 27. leprosy; 28. thalassemia; 29. intellectual disability (Down syndrome); 30. periodontal disease (gingivitis); 31. paresis; and 32. paralysis.

6 Unlike this study, Kirdruang’s study (2011) used the full sample without dividing those over age 18 and those under age 18; thus, we re-calculated to compare.
